# MicroRNA 486-3P as a stability marker in acute coronary syndrome

**DOI:** 10.1042/BSR20160023

**Published:** 2016-06-30

**Authors:** Tianling Wei, Lasse Folkersen, Ewa Ehrenborg, Anders Gabrielsen

**Affiliations:** * Department of Dermatology, Bispebjerg Hospital, Bispebjerg, Denmark; † Department of Systems Biology, Technical University of Denmark, Lyngby, Denmark; ‡ Atherosclerosis Research Unit, Department of Medicine, Center for Molecular Medicine, Karolinska University Hospital, Stockholm, Sweden; § Department of Cardiology, Karolinska University Hospital, Stockholm, Sweden

**Keywords:** coronary artery disease, diagnosis biomarker, microRNA

## Abstract

Easily accessible biomarkers are needed to diagnose cardiovascular disease precisely—particularly, to distinguish between disease subtypes that are encountered in clinical practice. Per the hypothesis that plasma miRNA is valuable for this purpose, we performed complete transcriptional profiling of an miRNA discovery-set in 14 samples: three patients with ST-elevated acute myocardial infarction (STEMI) at baseline and after three months of follow-up, four with stable ischaemic heart disease (stable-IHD) and four healthy age-matched volunteers. Our aim was to determine whether we could distinguish patients with unstable plaques from stable patients following a STEMI event. After analysing miRNA profiles, we conducted a validation study comparing three-month STEMI (*n*=40) with stable-IHD (*n*=35), which confirmed that *miR-486-3P* differentiates patients with three-month STEMI from those with stable-IHD (*P*=0.019).

## Abstract (precis)

A total of 75 cardiovascular patients were investigated for levels of the circulating *miR-486-3P*. The objective was to find out if any miRNA could distinguish patients with unstable plaque from patients with stable plaque after acute coronary syndrome.

## INTRODUCTION

Troponin (cTn) measurements are commonly used by clinicians to diagnose patients. cTn is generally accepted as the most reliable biomarker for the diagnosis of acute coronary syndromes. Despite its usefulness in the acute setting, cTn cannot distinguish between patients with stable coronary artery disease and patients at risk for coronary atherothrombosis in the general population [1]. Because treatment and prognosis differ significantly in these populations with coronary artery disease, it would be of interest to have a biomarker which could potentially separate patients at risk for atherothrombosis from patients with stable coronary artery disease.

In an attempt to further develop diagnosis and risk-stratification in patients with coronary artery disease, several novel biomarkers have been examined, typically in combination with cTn [1]. One notable class of biomarkers is miRNAs, which are approximately 22 nt noncoding RNAs that regulate gene expression at the post-transcriptional level. In the human genome, miRNAs constitute 1%–5% of all genes, making them the most abundant class of regulators. Although the function of most miRNAs is unknown, they have been shown to mediate various pathological conditions, including cardiovascular disease, and the levels of several miRNAs are altered in the heart in response to pathological stress.

miRNAs have properties that render them attractive biomarkers. They are remarkably stable in blood and can be specifically measured easily, which is typical of nucleotide-based markers [2]. These features have led to many miRNA biomarker candidates being reported [3], as reviewed in [4]. miRNA markers that have continued on to validation studies have garnered significant interest, such as *miR-499*, which has been shown to be an effective biomarker of acute myocardial infarction (AMI) in a Chinese population [5].

In our study, we aimed to identify miRNA markers that could distinguish patients stabilized following an acute coronary syndrome from patients with stable coronary artery disease. To this end, we measured the global expression of circulating miRNAs and assessed their diagnostic value as sensitive biomarkers of plaque stability in patients during the onset of and recovery from STEMI, i.e. atherothrombosis, patients who were undergoing elective percutaneous coronary intervention (PCI), i.e. stable ischaemic heart disease (stable-IHD), and healthy individuals. We focused primarily on patients at baseline and at three-month follow-up after ST-elevated acute myocardial infarction (STEMI), as this time point reflects a time where the acute atherothrombosis, myocardial injury and inflammatory response has passed but where the risk of renewed atherothrombotic events are elevated compared with patients with stable coronary artery disease. If, in the future, miRNAs demonstrate the ability to further risk-stratify and provide prognostic information they may become important clinical instruments.

## MATERIALS AND METHODS

### Patient populations

For the global circulating miRNA array, we recruited three STEMI patients 24 h–48 h after admission to the hospital (STEMI-0) and three months after discharge (STEMI-3). Four patients with stable coronary artery disease undergoing elective PCI (stable-IHD) and four healthy individuals were added as controls. For validation, a new cohort of 40 patients at three months after hospital discharge (STEMI-3) and 35 patients undergoing elective PCI (stable-IHD) were included (Table 1). All patients provided written informed consent. The study was approved by the Stockholm Regional Ethics Committee and conducted per the Declaration of Helsinki.

**Table 1:** Demographics of patients, stratified by cohort Demographics of patients, stratified by cohort. Categorical variables are reported as percentage (count). Numerical variables are reported as mean±S.D. The STEMI-3 discovery group was assumed to have unchanged variables for all values except statin treatment, cholesterol and triacylglycerols, for which no information was available. *P*-values for the validation cohort were calculated by Student's *t* test for numerical variables and chi-square test for categorical variables.

	Discovery cohort				Validation cohort		
	STEMI-0	STEMI-3	PCI	Healthy	STEMI-3	PCI	*P*-value
*n*	3	3	4	4	40	35	0.66
Male gender	100% (3)	100% (3)	100% (4)	100% (4)	76% (25)	84% (21)	0.059
Age (years)	48±3.6	48±3.6	52±3.6	52±2.6	52±6.9	55±3.9	0.22
Cholesterol (mM)	6.6±0.42	–	4.1±1.5	5.6±0.69	5.2±1	4.8±1.1	0.67
Triacylglycerols (mM)	2.8±1.4	–	1.1±0.46	1.3±0.49	1.9±1.3	2±1.4	0.03
Smoker	0% (0)	0% (0)	0% (0)	0% (0)	52% (17)	20% (5)	1.80×10^−6^
Statins	33% (1)	–	75% (3)	0% (0)	21% (7)	88% (22)	0.00035
β-Blockers	100% (3)	100% (3)	75% (3)	0% (0)	33% (11)	84% (21)	0.89
Waran	0% (0)	0% (0)	0% (0)	0% (0)	0% (0)	0% (0)	0.66

### Plasma sampling and RNA isolation

Blood samples were collected in ethylenediaminetetraacetic acid (EDTA)-K2 tubes and processed by two-step centrifugation (4°C at 820 × ***g*** for 10 min and 4°C at 16000 × ***g*** for 10 min). The supernatant was then transferred to RNase/Dnase-free tubes and stored at −80°C.

Total RNA was extracted from plasma using the CIAzol miRNeasy kit (Qiagen) with some modifications. Briefly, 200 μl of plasma was thawed on ice and lysed in 1 ml QIAzol lysis reagent. Samples in QIAzol were incubated at room temperature for 5 min to promote dissociation of nucleoprotein complexes. To adjust for variations in RNA extraction and co-purification of inhibitors, 5 μl of 5 nM synthetic *Caenorhabditis elegans* miRNAs, cel-*miR-39* miScript miRNA Mimic (Qiagen), was added to each sample. The remainder of the extraction was performed as per the manufacturer's instructions. Each sample was eluted in 20 μl RNase-free water and stored at −70°C.

### Low-density miRNA array

miRNAs were reverse-transcribed using the TaqMan® miRNA reverse-transcription kit with TaqMan miRNA Multiplex real time assays (human pool; Applied Biosystems). The real time PCR products were preamplified using the TaqMan PreAmp kit (Applied Biosystems), the products of which were diluted in 0.1X TE (Tris/EDTA) buffer and subsequently used as a template for miRNA expression profiling on TaqMan MicroRNA Low-Density Arrays (TLDAs) (Applied Biosystems).

For the initial screen, Human TaqMan miRNA microarrays (Card Av2.1 and Card Bv2.1, Applied Biosystems), covering 758 small, noncoding RNAs, were run on an ABI7900 HT with the TLDA upgrade. Raw data were analysed with SDS Relative Quantification, version 2.4.1, provided by Applied Biosystems. All measurements with *C*_t_ values > 35 or with a flag for issues according to the software were omitted (leaving 248, as plotted in Supplementary Figure S2). This threshold and quality filtering on low expression (*C*_t_ values > 35) left 201 miRNAs. Finally, miRNAs where only one sample in a group had a measurement passing quality control were omitted, leaving a final of 167758 miRNAs available for analysis.

For ΔΔ*C*_t_ normalization, the *C*_t_ of cel-*miR-39* was subtracted from that of the target, which was subtracted from the median of the calibrator (healthy individuals) and linearized to obtain 2∧−ΔΔ*C*_t_ values [6]. For the alternative Δ*C*_t_ normalization, the same procedure was followed, except for the subtraction of cel-*miR-39*. Plots of normalization values are shown in Supplementary Figure S1.

### Real-time PCR validation

Specific miRNAs by TaqMan® Real-Time PCR were quantified as per the manufacturer (Applied Biosystems). Target gene expression was normalized as described above for the low-density arrays (LDAs).

### Statistics

All *P*-values were calculated by two-sided Student's *t* test, assuming non-equal variance. In the validation step, outlier measurements outside of the 90% confidence interval were omitted from analysis to ensure normal distribution of the data. The validation measurements were distributed normally by Shapiro–Wilk test (*P*>0.05). *P*-values were uncorrected for multiple testing. The multiple testing burden of the discovery cohort was 201 tests, but independent samples were used in the validation cohort to avoid false discovery issues.

For testing of covariates, the following commonly used measures were investigated: sex, age, cholesterol, triacylglycerols, smoking, leucocytes, eosinophils, neutrophils, drug-eluting stents (DES), erythrocyte volume fraction, haemoglobin, thrombocytes, monocytes, statins, β-blockers, waran, diuretics, nonsteroidal anti-inflammatory drug (NSAID), acetylsalicylic acid (ASA), systolic blood pressure, diastolic blood pressure, pulse, prior heart infarction, prior angina, ejection fraction, weight, hip circumference, height and infection status. The association between *miR-486-3P* and validation sample group was re-calculated using each of these as a covariate in a linear regression model according the formula: miRNA ∼ group + covariate. The linear regression model was used as implemented in the *lm* function in R version 3.2.3.

## RESULTS

### miRNA profiles in serum of patients and healthy volunteers

Complete miRNA profiling was performed in 14 serum samples from patients with STEMI at baseline (STEMI-0) and after three months of follow-up (STEMI-3), patients with stable-IHD and healthy age-matched volunteers. A total of 758 miRNAs were measured on a LDA platform. After a filtration step for poor quality and low expression, 167 miRNAs remained for analysis.

Three normalization methods were tested before the miRNA profiles were analysed: (1) a *housekeeping gene* method, using the ΔΔ*C*_t_ method with U6 snRNA as an endogenous control; (2) a spike-in method, using the ΔΔ*C*_t_ method with measured values of cel-*miR-39*, which was spiked in before RNA purification; and (3) an miRNA mass method, using a ‘Δ*C*_t_’ method in which a comparable amount of total miRNA was assumed to exist in each measurement. The relative *C*_t_ values of each normalization factor are included as Supplementary Figure S1. Because the spike-in method with cel-*miR-39* demonstrated the least variation, it was chosen for further analysis.

### Discovery of differentially expressed miRNAs

For each measurable miRNA, we generated plots of their expression level as a function of patient group (Supplementary Figure S2). We aimed to identify biomarkers of plaque stability after acute myocardial infarction (MI), comparing global miRNA expression between STEMI-3 and stable-IHD patients. The plaque status of STEMI-3 patients was considered unstable. All 167 miRNA expression profiles were inspected manually, with a focus on strong differential expression between groups (S2). Extra weight was given to miRNA profiles with comparable expression between healthy individuals and stable-IHD patients.

**Figure 1:**
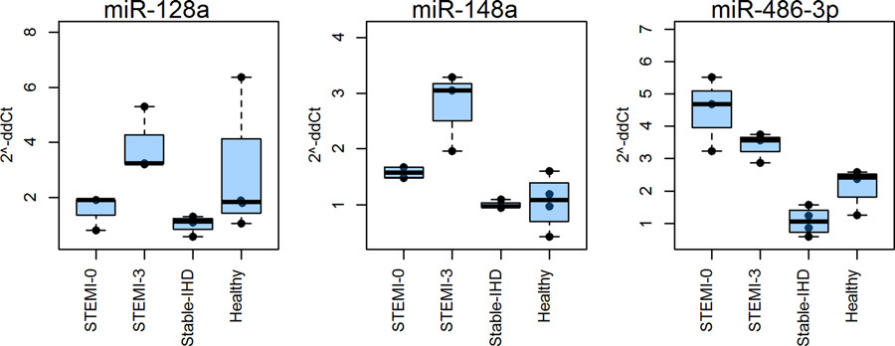
Expression profiles of miRNAs LDA-generated expression profiles of the three miRNAs chosen for further validation.

Based on this analysis, *miR-128a*, *miR-148a* and *miR-486-3P* (Figure 1) differentiated between patient groups. The levels of these species in STEMI-3 differed from the stable-IHD and healthy groups at magnitudes that had a realistic chance of being reproduced in a validation experiment. Unfortunately, the recently validated baseline AMI biomarker *miR-499* [5] had to be omitted for quality control reasons and thus was not tested in this cohort.

### Validation of differentially expressed miRNA

**Figure 2:**
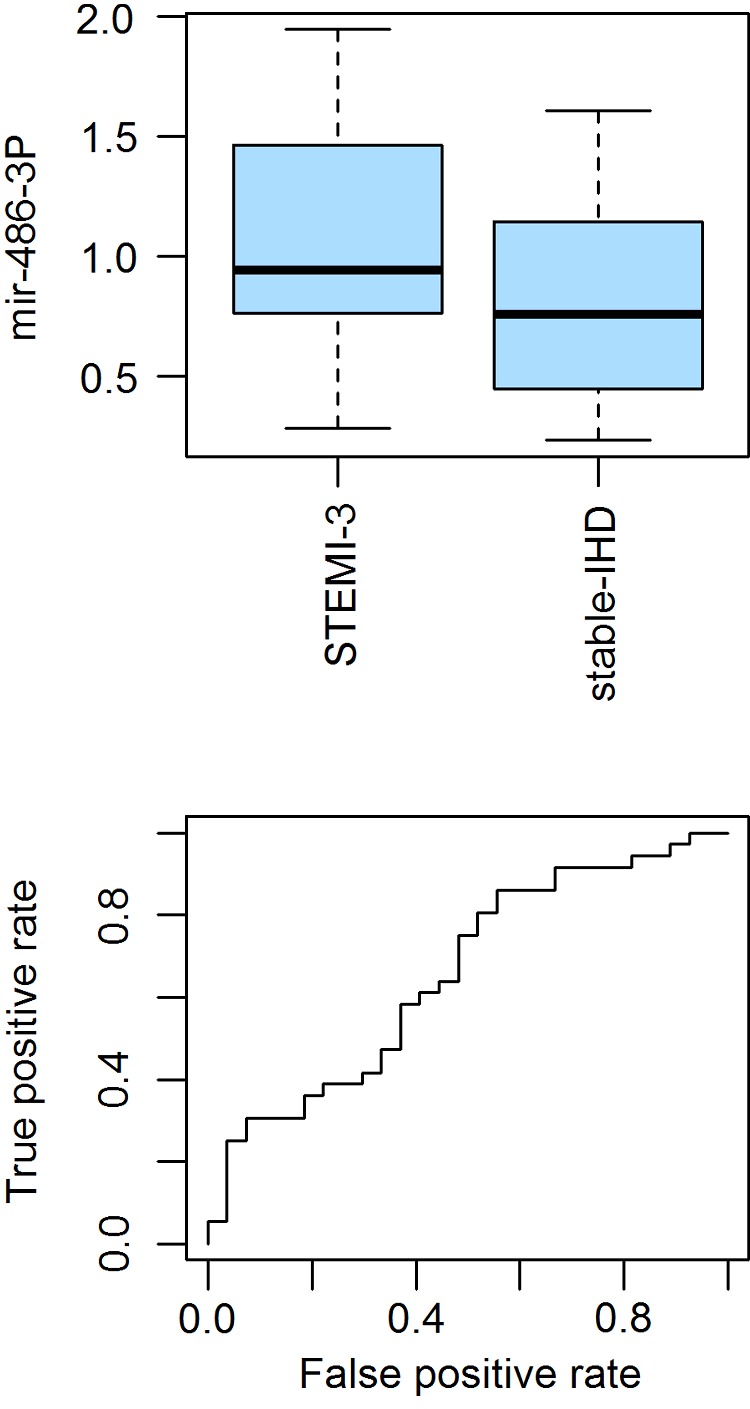
Biomarker validation data (**A**) Validation measurements in an independent cohort of STEMI-3 (*n*=40) and stable-IHD patients (*n*=35), in which *miR-486-3P* is significantly higher and at three months after an AMI event (*P*=0.0192). The *y*-axis shows 2∧−ΔΔ*C*_t_ values. (**B**) ROC curve of the predictive ability of *miR-486-3P* to distinguish STEMI-3 and stable-IHD patients.

Expression profiles were validated in the serum of 40 STEMI-3 patients and 35 patients from the stable-IHD group. *miR-486-3P* was significantly validated, with 1.31-fold higher expression in STEMI-3 compared with stable-IHD patients (*P*=0.019, Figure 2A). Further, *miR-148a* and *miR-128a* were not significant: *P*=0.085 and *P*=0.288 respectively, which excluded both from further analysis.

Based on the validation of *miR-486-3P* in an independent cohort, we estimated its predictive ability using receiver operating characteristic (ROC) curves. Based on the resulting thresholds, *miR-486-3P* correctly predicted the unstable type (STEMI-3) with 87% probability and was incorrect for 60% of patients with stable disease (stable-IHD) (Figure 2B).

In the validation cohort, we examined whether the differential expression of *miR-486-3P* was attributed to common confounders, such as age, gender, smoking, statins, low density lipoprotein (LDL) levels, triacylglycerol levels, cell counts, blood pressure, presence of DES and 20 other frequently measured clinical variables. The association between *miR-486-3P* and sample group remained significant after inclusion of each covariate (*P*<0.05), except for smoking which had a slight diminishing effect on the association of *miR-486-3P* and sample group (*P*=0.085). Thus, we conclude that the predictive value of *miR-486-3P* was largely independent of routine confounders, although smoking could be considered as a covariate of interest in future studies.

## DISCUSSION

The objective of our study was to identify miRNA-based biomarkers that could distinguish patients stabilized following an acute coronary syndrome from patients with stable coronary artery disease, such as patients who are undergoing elective PCI. Biomarkers which have larger variation in concentration levels between patient groups than within patient groups are suitable for this. We were prompted by our interest in allocating clinical resources toward patients with the highest risk of future adverse events. The detection of subtle, undetected STEMI would benefit this prioritization, constituting a patent, unmet medical need.

To this end, we developed a study on a discovery cohort, comprising array-based miRNA-wide profiling, followed by examination of a validation cohort by follow-up measurements of specific miRNAs. This design best balanced the cost of the expensive array-based method with the need for a sufficient sample size to overcome the burden of multiple testing.

From the discovery cohort, we selected three miRNAs that had a desirable profile with regard to our primary aim. These miRNAs were measured in a larger independent cohort, from whom the main finding of the present study was derived—that *miR-486-3p* reproducibly distinguishes patients three months after a STEMI from patients with stable coronary artery disease patients. Also, the effect was independent of other commonly measured clinical parameters. These properties, in addition to serum miRNA being stable and easy to measure, render *miR-486-3p* a potential marker for use in precision medicine. Notably, Hsu et al. [7] reported that *miR-486-3p* is differentially expressed between STEMI patients at baseline and healthy controls, further underscoring that *miR-486-3P* might have clinical value in STEMI. In line with Hsu et al. [7], we can also detect elevated plasma *miR-486-3P* at STEMI-0 compared with healthy controls (Figure 1). The injured myocardium could be a source for *miR-486-3P*. However, *miR-486-3P* is still detectable three months after suggesting that *miR-486-3P* has other origins outside the myocardium.

Although the predictive ability of *miR-486-3P* is at best modest, there are few predictive methods for late STEMI, and any ability to focus limited clinical resources might benefit a patient's quality of care. Similarly, much work is being performed to create multi-signature prediction profiles, and a novel biomarker from a nonstandard class has the potential to contribute additional clinical knowledge. Thus, our findings can effect more precise and data-driven clinical management.

## Abbreviations

AMI: acute myocardial infarction
ASA: acetylsalicylic acid
DES: drug-eluting stents
EDTA: ethylenediaminetetraacetic acid
LDA: low-density array
LDL: low density lipoprotein
MI: myocardial infarction
NSAID: nonsteroidal anti-inflammatory drug
PCI: percutaneous coronary intervention
ROC: receiver operating characteristic
stable-IHD: stable ischaemic heart disease
STEMI: ST-elevated acute myocardial infarction
TLDA: TaqMan MicroRNA Low-Density Array
Tn: troponin
